# Vitamin B12 Supplementation: Preventing Onset and Improving Prognosis of Depression

**DOI:** 10.7759/cureus.11169

**Published:** 2020-10-26

**Authors:** Prerna Sangle, Osama Sandhu, Zarmeena Aftab, Adarsh Thomas Anthony, Safeera Khan

**Affiliations:** 1 Family Medicine, California Institute of Behavioral Neurosciences & Psychology, Fairfield, USA; 2 Internal Medicine, California Institute of Behavioral Neurosciences & Psychology, Fairfield, USA

**Keywords:** serum vitamin b12, depression prevention, hyperhomocysteinemia, neuro-psychiatric

## Abstract

Depression is a common mental health condition occurring across all ages, genders, and populations and is almost always multifaceted. It can manifest as a form of metabolic disorder, endocrine disorder, cardiovascular diseases, inflammatory disorders, deficiencies, or neurodegenerative disorders. Although there have been various treatment options available for the treatment of depression, it is still a sizable global health concern requiring more attention. This review article was produced by researching data and studies to prove a relationship between Vitamin B12 and depression. Numerous studies were reviewed, and based on these studies, it was concluded that supplementation of Vitamin B12 early enough can delay the onset of depression and improve the effect of anti-depressants when used in conjunction with Vitamin B12. Although other vitamins like Vitamin B6 and folate are known to have an impact on depression, we have primarily focused on Vitamin B12 in an attempt to offer the providers a foundation to address this concern with their patients prone to depression or have had a major depressive episode in their life.

## Introduction and background

Over 264 million individuals suffer from depression across the world. Depression is one of the most commonly occurring mental health disorders occurring in adults and children [1]. The World Health Organization reported depression as one of the leading causes of disability worldwide [2].

**Figure 1:**
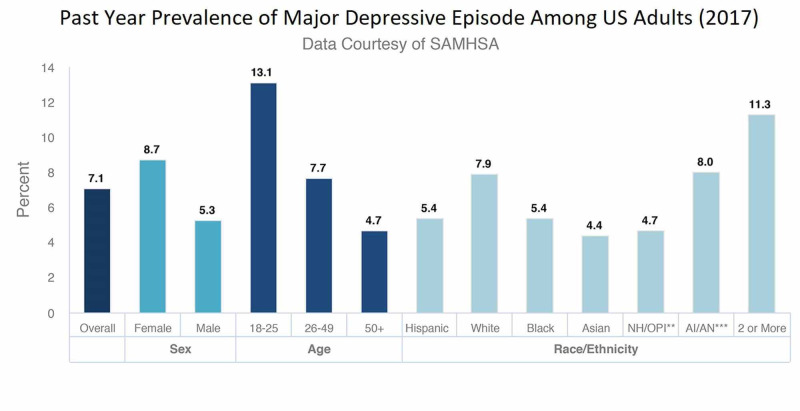
Prevalence of Major Depressive Episodes Among US Adults SAMHSA: Substance Abuse and Mental Health Services Administration; NH/OPI: Native Hawaiian/Other Pacific Islander; AI/AN: American Indian/Alaska Native

The American Academy of Pediatrics' most recent guidelines states that depression screening is recommended to begin at as low as 12 years of age. It is a well-known fact that depression can become a serious medical condition, if long-lasting, and can affect the quality of life, reduce overall productivity, and lead to extreme thoughts known as suicidal ideation [1]. The most known causes of depression are external factors like abuse, death or loss, heredity, and medications. Over 17.3 million US adults, about 7.1% of the total population, have suffered from a minimum of one major depressive episode in their life. Figure 1 shows the statistical data from 2017 outlining the prevalence of depression among adults in the United States.

Depression is more prevalent in women and young adults aged 18-25 years of age [3], most likely reported due to emotional or psychological stress. However, the completion of suicide attempts is more among men than women. 

Vitamin B12 is a water-soluble vitamin synthesized by the bacteria present in the human body. It is predominantly acquired by the intake of meat products, and thus individuals with a vegetarian or vegan diet tend to be naturally deficient. Deficiency of this particular vitamin can lead to fatigue, weakness, constipation, balance issues, mental fogginess, peripheral tingling, depression, and cognitive issues [4,5].

Numerous studies are being performed to determine the relationship between Vitamin B12 and depression, and if supplementation of Vitamin B12 can slow the progression of depression or prevent it. So far, there has not been concrete evidence that shows the positive effects of Vitamin B12 supplementation in patients with or prone to depression. However, some studies have shown positive effects on patients with depression when supplementation of Vitamin B12 is implemented. Some clinical studies have shown that higher Vitamin B12 levels in the body resulted in better outcomes in patients suffering from depressive and other mental health disorders [6], whereas some studies have also stated that adolescents with borderline levels of serum Vitamin B12 levels develop cognitive changes requiring treatment [7].

Depression is a lifelong battle for the majority of the population across the world. Especially with the current situation in the world and the COVID-19 pandemic associated with lockdowns, loss of jobs, housing, and immigration statuses, the incidence of depression and other mental health disorders has significantly increased among children, adolescents, and adults, not being limited to a specific age group. This paper aims to find the effects of Vitamin B12 on depression with the help of other reviewed articles, in an attempt to allow providers to make evidence-based decisions to prevent and manage depression by merely supplementing individuals with Vitamin B12.

## Review

Our review focuses on the effects of Vitamin B12 supplementation at the onset or during the prognosis of depression. A total of 35 studies and articles were reviewed for this review [1-35]. We used PubMed and Google Scholar as our main databases to identify, screen, and choose the relevant articles. We chose the studies that were relevant to the topic and were published in English. We did not include gray literature or unpublished studies.

Vitamin B12 and hyperhomocysteinemia

Micronutrients are known to affect the normal structure and functions of the brain. For this paper, we will be focusing on one specific micronutrient - Vitamin B12. Cyanocobalamin, also known as Vitamin B12, is one of the most important water-soluble vitamins. The primary functions of Vitamin B12 are appropriate red blood cell formation, neurological functioning, and DNA synthesis [8]. Deficiency of Vitamin B12 can result in hematological changes, neurological and psychiatric problems, which can manifest as irritability, changes in personality, depression, and memory loss [9]. It is also known to worsen depression by excitotoxic reactions caused by the accumulation of homocysteine [10].

Vitamin B12 deficiency is reportedly known to cause mental disturbances in numerous individuals. However, there is little evidence to show the correlation between psychosis and Vitamin B12. Cobalamin is one of the essential elements for monoamine neurotransmitter synthesis in the brain. A study published in 2011 in the Academy of Psychosomatic Medicine included supplementation of Vitamin B12 and folate, which resulted in improved cognitive functioning among patients participating in the study. A borderline Vitamin B12 level of 75-95 pg/mL, as well as up to a normal level of 307 pg/mL, is known to cause neuropsychiatric symptoms in patients, and such symptoms may start manifesting even with such borderline deficiencies or low normal levels of Vitamin B12. The study does dispute if the effects were of the Vitamin B12 supplementation or anti-depressant medications; however, it concludes that supplementation did increase the overall response to the anti-depressants [11].

Hyperhomocysteinemia

**Figure 2:**
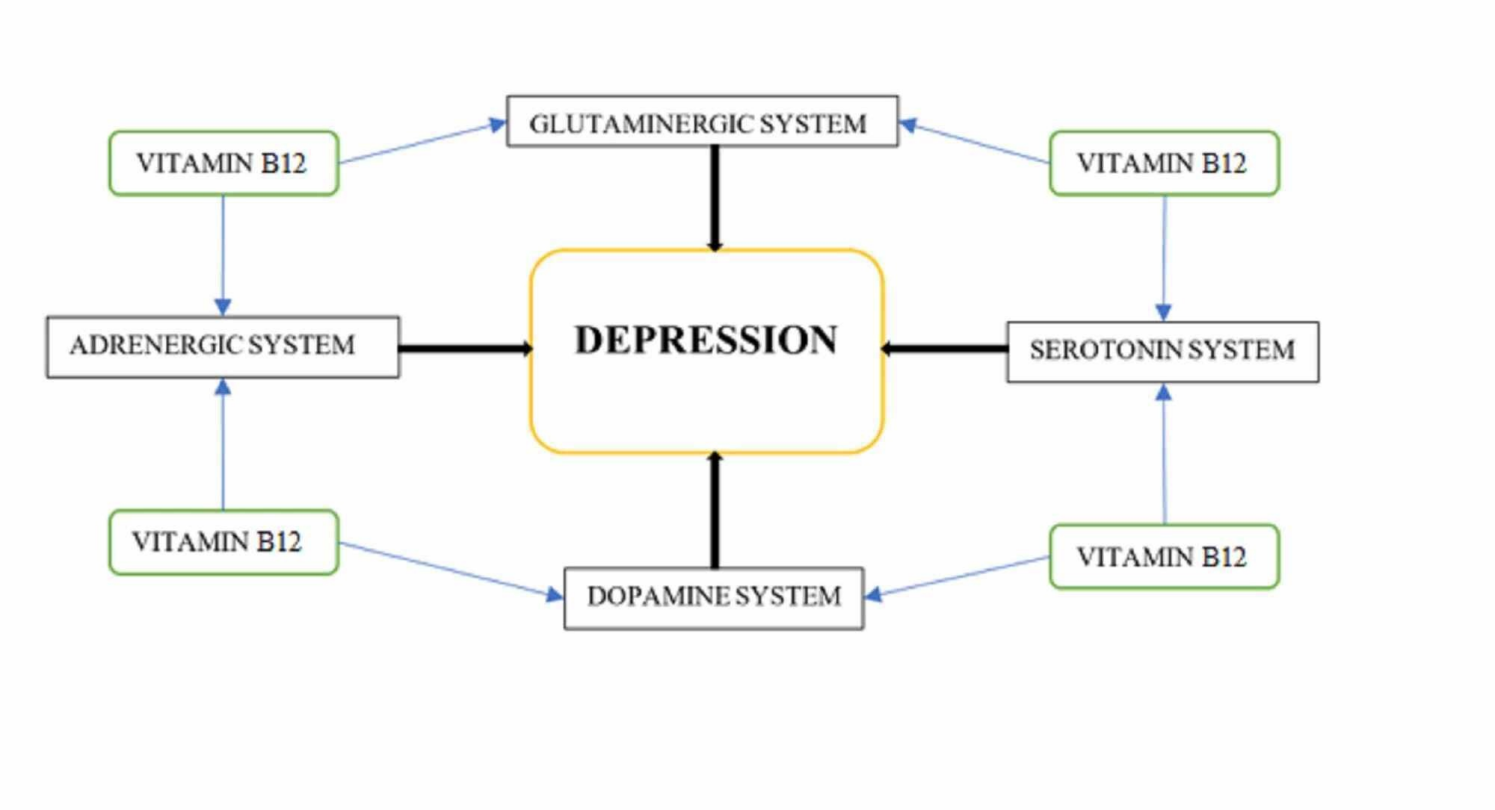
Neurochemical Pathways Affected by Vitamin B12 Levels

Higher homocysteine levels are associated with a phenomenon called "methionine loading." A deficiency of Vitamin B12 along with B6 and folate usually prevents the conversion of homocysteine to methionine, increasing the levels of homocysteine. Hyperhomocysteinemia can also occur in patients with renal disorders or genetic alterations of methyl-tetrahydro-folate reductase or cystathionine beta-synthetase, which are required for the metabolism of homocysteine [12,13]. Higher levels of homocysteine affect the DNA formation and overall turnover of red blood cells (RBCs), causing the development of megaloblastic or pernicious anemia, ultimately affecting the cognitive ability and mood of the patient [14-17]. Figure 2 gives an overview of the various neurochemical pathways affected by Vitamin B12 levels, which ultimately leads to some form of depressive disorder or episode.

Vitamin B12 is interdependent with other micronutrients, mainly Vitamin B6 and folate. This interdependence affects multiple neuronal pathways that are not directly controlled by Vitamin B12. Neurotransmitters are required to conduct the signals from one neuron to another, with the help of pre- and post-synaptic junctions. The malfunctioning of any of these pathways can lead to depression [4]. Lower Vitamin B12 levels are also known to increase the risk of cognitive decline, Alzheimer's disease, and dementia. According to Vogiatzoglou et al., higher Vitamin B12 levels have proven to protect patients against brain atrophy [18].

Pathophysiology of depression

Over a decade ago, depression was commonly seen in adult and elderly patients but had become far more common among the younger population. According to the Journal of Psychological Medicine, the rates of depression have increased drastically among children as young as 12 years and older.

Depression, once diagnosed, is clinically treated with anti-depressants and cognitive behavioral therapy (CBT). In patients where the anti-depressant regimen is not eliciting a response, an additional drug from a different pharmaceutical group has proved to have a significant effect [19,20]. However, long-term and extensive pharmacotherapy are almost always associated with the extent of undesirable effects on the patient like the risk of major adverse cardiovascular events, decline in cognitive functioning over time, behavioral changes including but not limited to sexual problems, and feeling of "emotional numbness." The B vitamins are crucial particulates of the 1-carbon metabolism wherein 5-methyltetrahydrofolate and methylcobalamin help convert homocysteine to methionine, which in turn forms S-adenosylmethionine (SAM), which is the essential methyl donor required for the production of monoamine neurotransmitters, phospholipids, and nucleotides [21].

A study performed in 2010 by Skarupski et al. included a sample US population of 35,053 older adults, and the results of this study stated that higher intakes of Vitamin B12 and B6 were associated with a lower risk of developing depressive symptoms by an average of 7.2 years [23]. According to Hutto, cobalamin and folate are components of the synthesis of monoamine neurotransmitters, which may lead to psychotic behavior by a rise in BH4 (tetrahydrobiopterin) production [24]. Moorthy et al. published an article in the *Journal of Nutrition* in 2010, which discussed the C677T polymorphism of the methylenetetrahydrofolate reductase (MTHFR) gene in various ethnic groups. The study showed lesser scores on the Mini-Mental Status Exam (MMSE) and higher depression scores among patients with lower plasma levels of Vitamin B12 [25].

Daily supplementation of oral Vitamin B12 (100 mcg) and folic acid (400 mcg) has been shown to increase cognitive function in a randomized controlled trial performed by Walker et al. in 2012 [26]. They proposed two theories by which this could occur: reduced homocysteine levels and lowered vascular and metabolic risk factors occurring due to supplementation with Vitamin B12 and folic acid [26]. Another study performed in 2012, specifically among the Iranian population with major depressive disorders, also indicated lower plasma concentrations of Vitamin B12 among the Tabrizian patients who had depressive symptoms [27]. 

**Figure 3:**
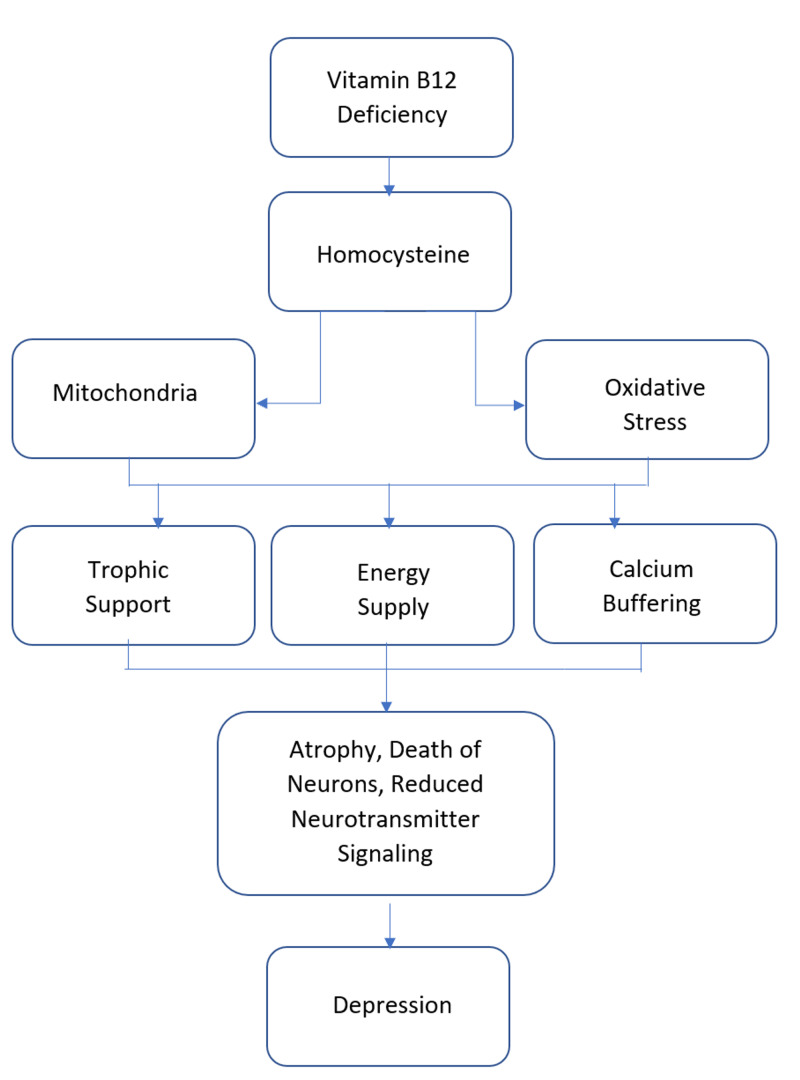
Pathophysiology of Depression

Melancholic depressive symptoms were more relatively associated with lower plasma Vitamin B12 levels. One such study reviewed concluded that Vitamin B12 deficiency played an important role in the pathogenesis of depressive symptoms [28]. A randomized controlled trial performed in 2013 among the Pakistani population attempted to identify the coexistence of depression and Vitamin B12 deficiency. This study reported that 22% of the sample depressed population had associated low Vitamin B12 levels to constitute a deficiency, 36% had low normal Vitamin B12 levels, and 42% of the sample population had normal serum B12 levels [29]. Vitamin B12, in combination with folic acid, is known to reduce the levels of homocysteine, thereby reducing the toxic effects and thus improving the effect of anti-depressants by increasing the effectiveness of anti-depressants S-adenosylmethionine (SAMe) [30]. A study performed among the Indian population in 2017 demonstrated that Vitamin B12-deficient individuals, mostly vegetarians, were more prone to developing neurological problems [31]. Figure 3 illustrates the effects of Vitamin B12 deficiency on depression. 

The total cyanocobalamin and methylcobalamin levels in patients with autism and schizophrenia were reported to be lower as compared to the age-matched control subjects. Although the association with the levels and depression was not performed, the study analyzed brain samples [22]. It concluded Vitamin B12-dependent methylation reactions in the brain played a crucial role in the presentation of neurological symptoms and disorders [22]. A cross-sectional study was performed in 2018 to understand the association between vitamins and depression to gather data more based explicitly on sex-specific criteria; evidently, females were identified to be more at risk as compared to men and had coinciding lower serum Vitamin B12 levels [32]. Low normal serum Vitamin B12 is also posing a risk to pregnant women and makes them more prone to develop depressive symptoms. According to a secondary analysis of the National Health and Nutrition Examination Survey and a reported Patient Health Questionnaire-9 (PHQ-9) score of 10 or more among the subjects, pregnant women were reported to be 3.82 times more predisposed to developing depression [33].

One-carbon metabolism has been extensively linked with psychiatric disorders among numerous studies. One such recent study published in 2020 comprised of 89 patients, mainly children and adolescents, and included a patient group that was already diagnosed with depression and a control group. They were subsequently tested for serum levels of Vitamin B12, homocysteine, and Vitamin D. The study utilized correlation analysis and showed a negative relationship between the severity of depressive symptoms and Vitamin B12, and associated hyperhomocysteinemia, concluding that low normal levels or deficiency of Vitamin B12 can become a significant contributing factor toward depression [34]. Case-control studies performed have yielded similar results; however, the sample population has been limited, which necessitates studies comprising larger population groups. Patients diagnosed with depression are highly likely to be non-compliant, thus suggesting better ways of managing and overcoming the non-compliant behavior, which also goes hand in hand with improper dietary intake of essential nutrients. Khosravi et al. performed a case-control study to identify the correlation between increased and decreased intake of Vitamin B12 and folate with depression and its severity. An inversely proportional relationship was established between Vitamin B12 and folate levels and depression [35]. Table 1 gives a snapshot of the studies reviewed for this article along with their results. 

**Table 1:** Effects of Vitamin B12 on Depressive Symptoms SSRI: Selective serotonin reuptake inhibitor

Author	Year of Publication	Purpose of the Study	Results/Conclusion
Skarupski et al. [23]	2010	To determine if Vitamin B12 or B6 intake affected the onset of depressive symptoms	Higher intakes of both Vitamins B12 and B6 reduced depressive symptoms over time.
Walker et al. [26]	2012	To identify if folic acid and Vitamin B12 supplementation prevented cognitive decline	Long-term supplementation improved cognitive functioning
Gargari et al. [27]	2012	To assess nutritional status in patients with major depressive disorders	Vitamin B12 and folate levels were markedly lower among identified patients
Seppälä et al. [28]	2013	To identify a relationship between Vitamin B12 levels and depressive symptoms	Vitamin B12 levels were reported to be correlated with melancholic depressive symptoms more than non-melancholic.
Syed et al. [29]	2013	To compare the response of SSRI monotherapy vs. B12 augmentation in patients with depression	Supplementation of Vitamin B12 along with anti-depressant therapy greatly improved depressive symptoms.
Peppard et al. [33]	2019	To identify the risk of depression in pregnant women	The study stated that women during the antenatal period with low normal Vitamin B12 levels were 3.82 times more prone to develop depression.

Among the numerous articles reviewed for this topic, the majority of them proposed that Vitamin B12 levels are related to the severity of depression. Although this topic lacks tangible and sizable data to prove the same, healthcare providers need to take into consideration the studies reviewed in this paper and utilize them to test the effects of Vitamin B12 supplementation in patients with neuropsychiatric disorders.

Limitations

The limitations of this topic are that the studies that have been reviewed for this paper have had relatively smaller sample population sizes. There is still a need for more research with a larger sample size to find more concrete evidence of the positive effects of Vitamin B12 on depression.

## Conclusions

In this article, we reviewed over 30 to 40 published articles and studies to understand the correlation between the supplementation of Vitamin B12 and the prognosis of depression. Based on the reviewed studies, it was found that although there is no concrete evidence showing positive effects of Vitamin B12 on depression or depressive symptoms, the lower levels of Vitamin B12 in the body are associated with a higher risk of developing depression. Routine testing for plasma Vitamin B12 levels can be recommended for patients across the board beginning at the adolescent age to prevent the population from developing depression or other forms of cognitive under-functioning, which may lead to depression. Low normal levels of Vitamin B12 should be recommended to be evaluated for symptoms of Vitamin B12 deficiency, including any neurological manifestations, as these symptoms can present even when the levels are within a normal range. However, additional studies with larger population groups should be done to formulate an evidence-based criterion for providers to adapt across the world.
